# Severity scores for status epilepticus in the ICU: systemic illness also matters

**DOI:** 10.1186/s13054-022-04276-7

**Published:** 2023-01-16

**Authors:** Fang Yuan, Charlotte Damien, Nicolas Gaspard

**Affiliations:** 1 Neurology Department, The Second Affiliated Hospital of Guangzhou University of Chinese Medicine, Guangzhou, China; 2 Service de Neurologie, Hôpital Universitaire de Bruxelles, Hôpital Erasme, Université Libre de Bruxelles, 1070 Brussels, Belgium; 3 Neurology Department, Yale University School of Medicine, New Haven, CT USA

**Keywords:** Status epilepticus, Mortality, Critical care, Severity scores, Systemic illness

## Abstract

**Background:**

Current prognostic scores for status epilepticus (SE) may not be adequate for patients in ICU who usually have more severe systemic conditions or more refractory episodes of SE. We aimed to compare the prognostic performance of two SE scores, Status Epilepticus Severity Score (STESS) and Epidemiology-Based Mortality Score in Status Epilepticus (EMSE) score, with four systemic severity scores, Acute Physiology and Chronic Health Evaluation 2 (APACHE-2), Simplified Acute Physiology Score 2 (SAPS-2), Sequential Organ Failure Assessment (SOFA) score, and Inflammation, Nutrition, Consciousness, Neurologic function and Systemic condition (INCNS) score in critically ill patients with SE.

**Methods:**

This retrospective observational study of a prospectively identified SE cohort was conducted in the ICU at a tertiary-care center. The area under the receiver operating characteristic curve (AUC), sensitivity, specificity, positive predictive value, negative predictive value, accuracy, and associations with outcomes of STESS, EMSE, INCNS, APACHE-2, SAPS-2, and SOFA score for the prediction of in-hospital mortality and no return to baseline condition were assessed.

**Results:**

Between January 2015 and December 2020, 166 patients with SE in ICU were included in the study. In predicting in-hospital death, APACHE-2 (0.72), SAPS-2 (0.73), and SOFA score (0.71) had higher AUCs than STESS (0.58) and EMSE (0.69). In predicting no return to baseline condition, the AUC of APACHE-2 (0.75) was the highest, and the AUC of INCNS (0.55) was the lowest. When the specificity approached 90%, the sensitivity values of these scores were not quite acceptable (< 40%). Neither SE scores nor systemic severity scores had desirable prognostic power. In the multivariate logistic regression analyses, the best combinations of scores always included at least one or more systemic severity scores.

**Conclusions:**

STESS and EMSE were insufficient in outcome prediction for SE patients in ICU, and EMSE was marginally better than STESS. Systemic illness matters in ICU patients with SE, and SE scores should be modified to achieve better accuracy in this severely ill population. This study mostly refers to severely ill patients in the ICU.

**Graphical abstract:**

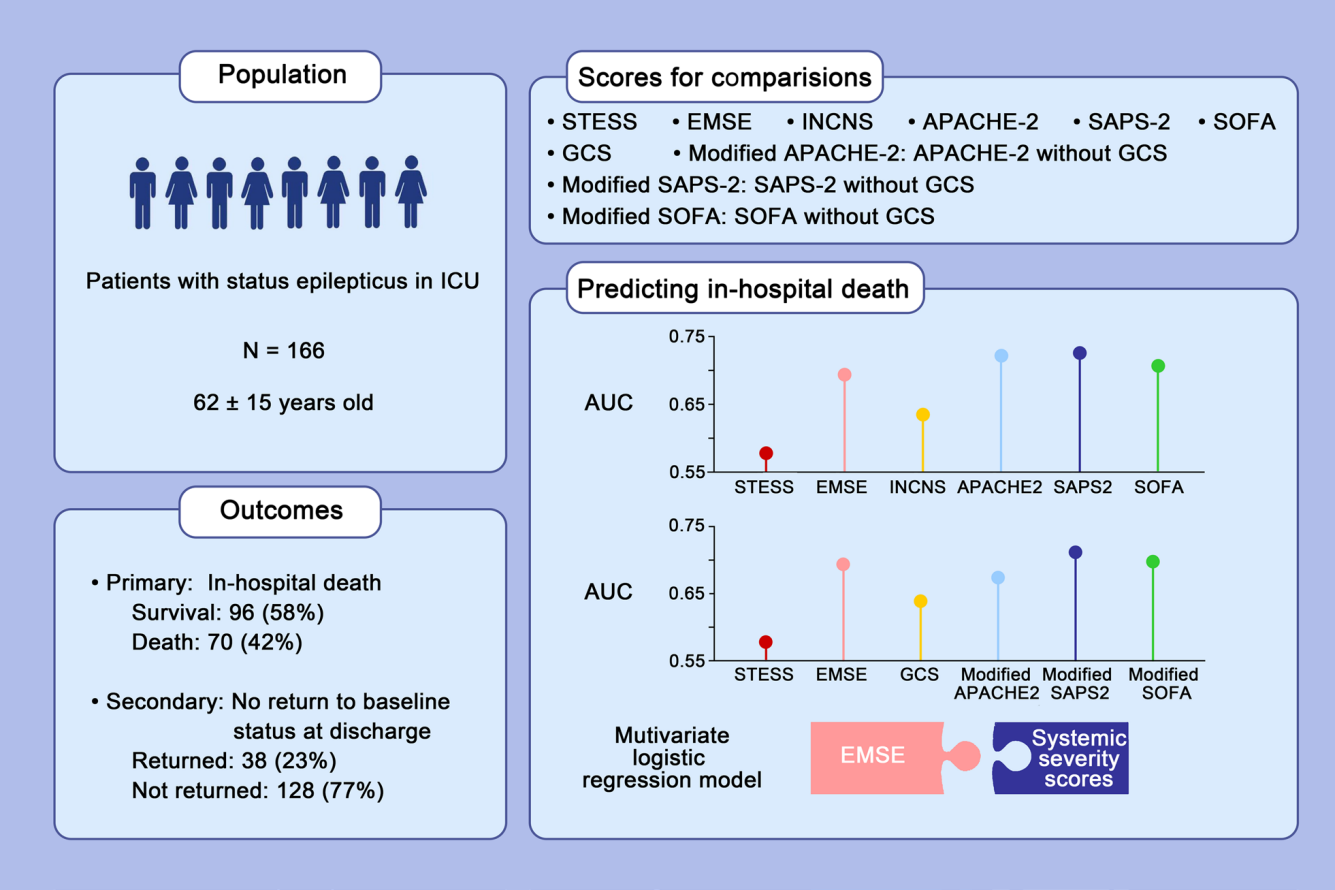

**Supplementary Information:**

The online version contains supplementary material available at 10.1186/s13054-022-04276-7.

## Introduction

Compared to the general population of patients with status epilepticus (SE), those admitted in an ICU typically suffer from more refractory episodes, a higher rate of systemic complications, such as infections, hypotension requiring vasopressors, and organ dysfunction, and a higher mortality of 17–67% [1–3]. Treating SE in critically ill patients is challenging as, on the one hand, SE is more often subtle and refractory [2, 4], and on the other hand, excessive and aggressive treatments are associated with longer duration of mechanical ventilation, more complications, and higher mortality in case of organ dysfunction [5–8]. Multimodal monitoring of neurological and systemic function, tailored anti-epileptic treatments, and advanced organ support are usually required for these patients [9–11]. An accurate severity scoring system for critically ill patients with SE is thus needed to individualize care and improve observational and interventional research.

Currently, both SE-specific and general illness severity scoring systems exist. The Status Epilepticus Severity Score (STESS) and Epidemiology-Based Mortality Score in Status Epilepticus (EMSE) are the most widely used scores for status epilepticus [12, 13]. However, their performance in ICU patients is questionable, with area under the receiver operating characteristic curve (AUC) ranging from 0.57 to 0.74 for STESS and from 0.53 to 0.64 for EMSE [14–16]. In particular, the STESS is known to overestimate mortality in patients with SE admitted to an ICU [17]. None of these scores incorporate measures of organ dysfunction beyond the brain so that more general, and complex, scores used to measure the severity of critical illness, might more accurately characterize critical illness in patients with SE, as they account for systemic organ dysfunction. A recent study comparing the STESS to the Acute Physiology and Chronic Health Evaluation-2 (APACHE-2) [18], the Simplified Acute Physiology Score-2 (SAPS-2) [19] and the sequential organ failure assessment (SOFA) score [20] found no additional value for these more complex scoring systems for the prediction of in-hospital death and functional outcome and suggested that the combination of the STESS and the Glasgow Coma Scale (GCS) was sufficient [21]. However, illness severity in this study was relatively low for an neurocritical care population, with only 17% patients requiring vasopressors and a mortality rate of 9%. Finally, the Inflammation, Nutrition, Consciousness, Neurologic function and Systemic condition (INCNS) score is a more recent score, developed to measure the severity of illness in neurocritical care patients [22], but it has never been investigated in SE.

Our aim was thus to assess and compare the prognostic performance of STESS, EMSE, APACHE-2, SAPS-2, SOFA, and INCNS for in-hospital mortality and return to baseline function in critically ill patients with SE.

## Methods

### Design

This retrospective observational study of a prospectively identified cohort was conducted in the ICU of Erasme Hospital, a tertiary teaching hospital of Université Libre de Bruxelles, Belgium. The Ethics Committee of Erasme Hospital approved this study (No.2020/103; Status Epilepticus Outcome Project; 02/11/2020). Informed consent to the participation in this study was waived. This study was conducted in accordance with the Declaration of Helsinki and its later amendments and was reported based on the STROBE guidelines [23].

### Patients

All consecutive adult patients (≥ 18 years old) diagnosed with SE and with an ICU stay ≥ 24 h between January 1, 2015, and December 31, 2020, were included. Patients with post-anoxic SE (a very specific subtype of SE with different electroclinical features and high mortality [24]) or missing data for all severity scores were excluded. Status epilepticus was defined according to published guidelines [25, 26]. All SE patients were monitored with bedside video-EEG with an array of 21 scalp electrodes for at least 24 h. All patients received evaluation, diagnosis, and treatment consultations of SE from the same team of neurologists and followed the same institutional treatment protocol derived from available recommendations [27, 28] .

### Data collection

Data were retrospectively collected from digital medical records and our EEG database. Patients’ demographics, SE features (seizure history, etiology, types, treatments), comorbidities (quantified by the Charlson Comorbidity Index [CCI]), and all the other laboratory and clinical findings required for the assessments of STESS, EMSE-EACE, APACHE-2, SAPS-2, SOFA, and INCNS score were collected. The worst value of each score within 24 h after ICU admission was used in this study. Clinical neurological evaluations required for APACHE-2, SAPS-2 and INCNS, such as Glasgow Coma Scale (GCS), muscle strength score, pupillary light reflex, corneal reflex, and swallowing function, were prospectively performed by qualified neurologists, intensive care physicians or nurses, as these evaluations are part of the routine bedside assessments and reported in the medical records.

### Outcomes

The primary outcome was in-hospital death. The secondary outcome was lack of return to baseline functional status at discharge, defined as any decrease in the estimated modified Rankin Scale (mRS) score at hospital discharge compared to the pre-admission mRS score, including death. The mRS score was retrospectively assessed by a trained neurologist who was blinded to all severity scores.

### Statistical analysis

Continuous variables were presented as mean (standard deviation, SD) or median (interquartile range, IQR), as appropriate, and categorical variables were presented as count (percentage). The discriminative power of STESS, EMSE-EACE, APACHE-2, SAPS-2, SOFA, and INCNS score was first assessed using the area under the ROC curves (AUC). The cutoff value of each score was determined by the Youden index. The sensitivity, specificity, positive predictive value (PPV), negative predictive value (NPV), and accuracy of these six scores at corresponding cutoff values were analyzed and compared. The McNemar test was used to compare sensitivity, specificity, and AUC between scores [29], and the modified Wald test was used to compare PPV and NPV [30]. We also investigated the sensitivity and accuracy of the scores at cutoff values that minimize the risk of false prediction (defined as specificity close to 90%), given the risk associated with premature withdrawal of life support therapies due to perceived poor prognosis.

In order to determine the discriminative power of systemic illness only (i.e., without the severity of the neurological illness), the AUCs and optimal cutoff for of modified APACHE-2 (APACHE-2 without GCS), modified SAPS-2 (SAPS-2 without GCS), and modified SOFA (SOFA without GCS), and the GCS alone were also assessed. Next, we examined predictive values of STESS, EMSE, and systemic severity scores using logistic regression analyses. Two models of combinations were assessed: STESS + EMSE + INCNS + APACHE-2 + SAPS-2 + SOFA (Model 1) and STESS + EMSE + GCS + modified APACHE-2 + modified SAPS-2 + modified SOFA (Model 2). Stepwise selection approaches were used in the multivariate logistic regression analyses to determine the best combinations that mostly correlated with the predefined outcomes. Statistical analyses were conducted using SPSS (version 22.0) and R (version R4.1.0). Statistical thresholds were adjusted for multiple comparisons, using the Bonferroni correction [31] .

### Data availability and role of the funding source

The deidentified data will be made available for non-commercial purposes with the approval of the corresponding author. The Fonds Erasme pour la Recherche Médicale supported this study but had no role in the study design, data interpretation, statistical analysis, or writing.

## Results

**Fig. 1:**
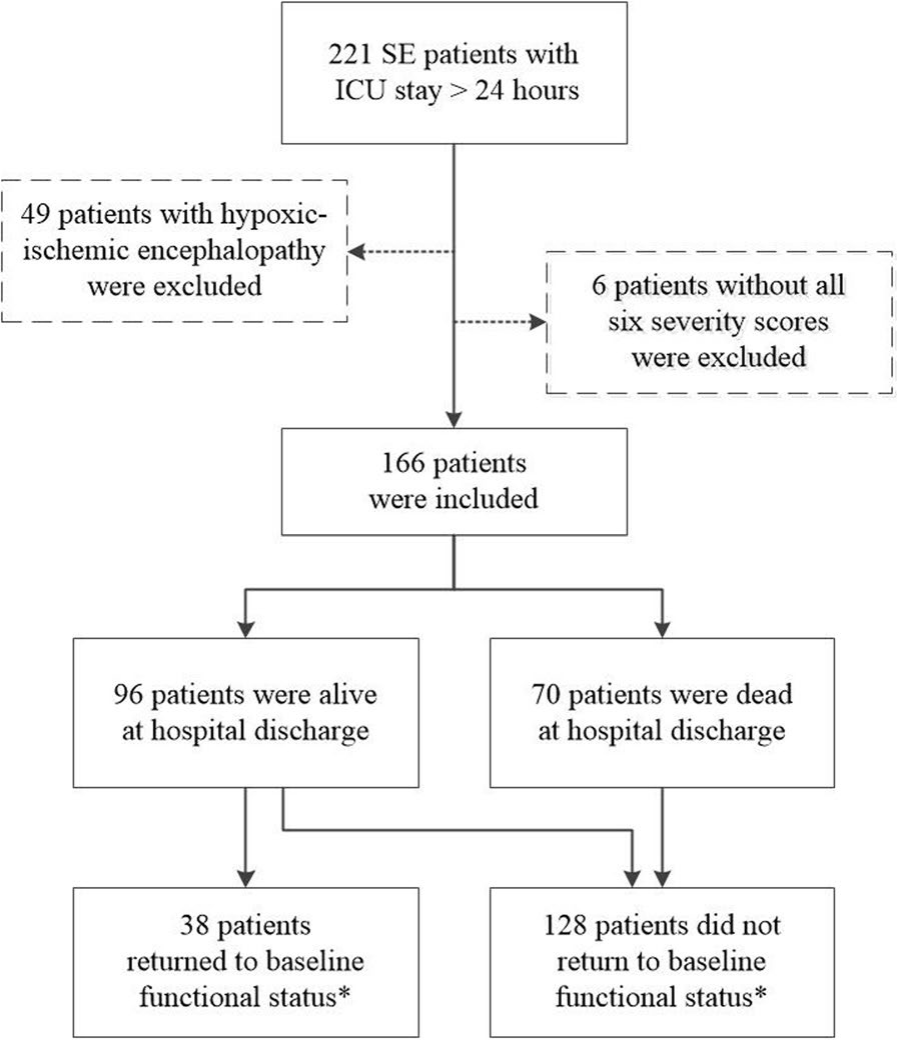
Flowchart. *Functional status was assessed using modified Rankin Scale

Among 221 SE patients with ICU stay > 24 h, 49 patients with post-anoxic SE and 6 patients without all six severity scores were excluded. Thus, 166 patients were included in the analysis (Fig. 1). Fifty-five (33%) patients died in the ICU, 99 (60%) patients were treated in the wards of our hospital before discharge, 5 (3%) patients were discharged from our ICU to other hospitals, 4 (2%) patients were discharged to rehabilitation centers, and 3 (2%) patients were discharged directly to the home. The median time between ICU discharge and final hospital discharge was 4 days (IQR, 0–18 days). At hospital discharge, 70 (42.2%) patients had died, and 128 (77.1%) patients did not return to baseline functional status. Demographics and clinical characteristics are presented in Table 1. Mean age was 62 ± 15 years, 50% were female, and the leading causes for SE were acute symptomatic etiologies (68.7%), including acute brain injury (34.9%) and acute medical conditions (33.7%). One hundred and thirty-seven (83%) patients had SE diagnosed in the ICU, and 29 (17%) patients had SE diagnosed outside the ICU. SE was refractory in 99 (60%) patients and super-refractory in 19 (11%) patients. Patients had multiple comorbidities and organ impairments, with a median Charlson Comorbidity Index (CCI) of 4 and a median SOFA score of 8.

**Table 1:** Clinical characteristics

Participant characteristic	*N* = 166
Age, years, mean ± SD	62 ± 15
Female, *n* (%)	83 (50.0)
Etiology, *n* (%)	
Acute symptomatic^a^	114 (68.7)
Remote symptomatic	33 (19.9)
Progressive symptomatic	10 (6.0)
SE in defined electroclinical syndromes	1 (0.6)
Unknown	8 (4.8)
Seizure history, *n* (%)	49 (29.5)
SE diagnosed in ICU, *n* (%)	137 (82.5)
SE diagnosed outside ICU, *n* (%)	29 (17.5)
Type of SE, *n* (%)	
With prominent motor symptoms	96 (57.8)
Convulsive SE	63 (38.0)
Myoclonic SE	11 (6.6)
Focal motor	22 (13.2)
Without prominent motor symptoms	70 (42.1)
Generalized NCSE with stupor or coma	30 (18.0)
Focal NCSE without coma	40 (24.1)
Refractory SE, *n* (%)	99 (59.6)
Super-refractory SE, *n* (%)	19 (11.4)
Baseline scores, median (IQR)	
GCS	6 (3–9)
CCI	4 (3–6)
STESS	3 (2–4)
EMSE-EACE	59 (35–84)
INCNS	16 (12–21)
APACHE-2	22 (18–26)
SAPS-2	56 (43–66)
SOFA	8 (5–11)
Treatments	
Number of antiseizure drugs, median (IQR)	3 (2–4)
Use of any CIVAD, *n* (%)	58 (34.9)
Use of ≥ 2 CIVADS, *n* (%)	16 (9.6)
Mechanical ventilation, *n* (%)	118 (71.1)
Use of vasopressors, *n* (%)	73 (44.0)

*APACHE-2* Acute Physiology and Chronic Health Evaluation-2, *CCI* Charlson Comorbidity Index, *CIVAD* continuous infusion of intravenous anesthetic drug, *EMSE-EAC* Epidemiology-based Mortality Score in Status Epilepticus-Etiology, Age, level of Consciousness, Electroencephalogram, *GCS* Glasgow Coma Scale, *INCNS* Inflammatory Nutritional Consciousness Neurological and Systemic Status, *IQR* interquartile range, *NCSE* nonconvulsive status epilepticus, *SAPS-2* Simplified Acute Physiology Score-2, *SE* status epilepticus, *SOFA* Sequential Organ Failure Assessment, *STESS* Status Epilepticus Severity Score

^a^Includes 58 patients with acute brain injury and 56 patients with acute medical condition

**Fig. 2:**
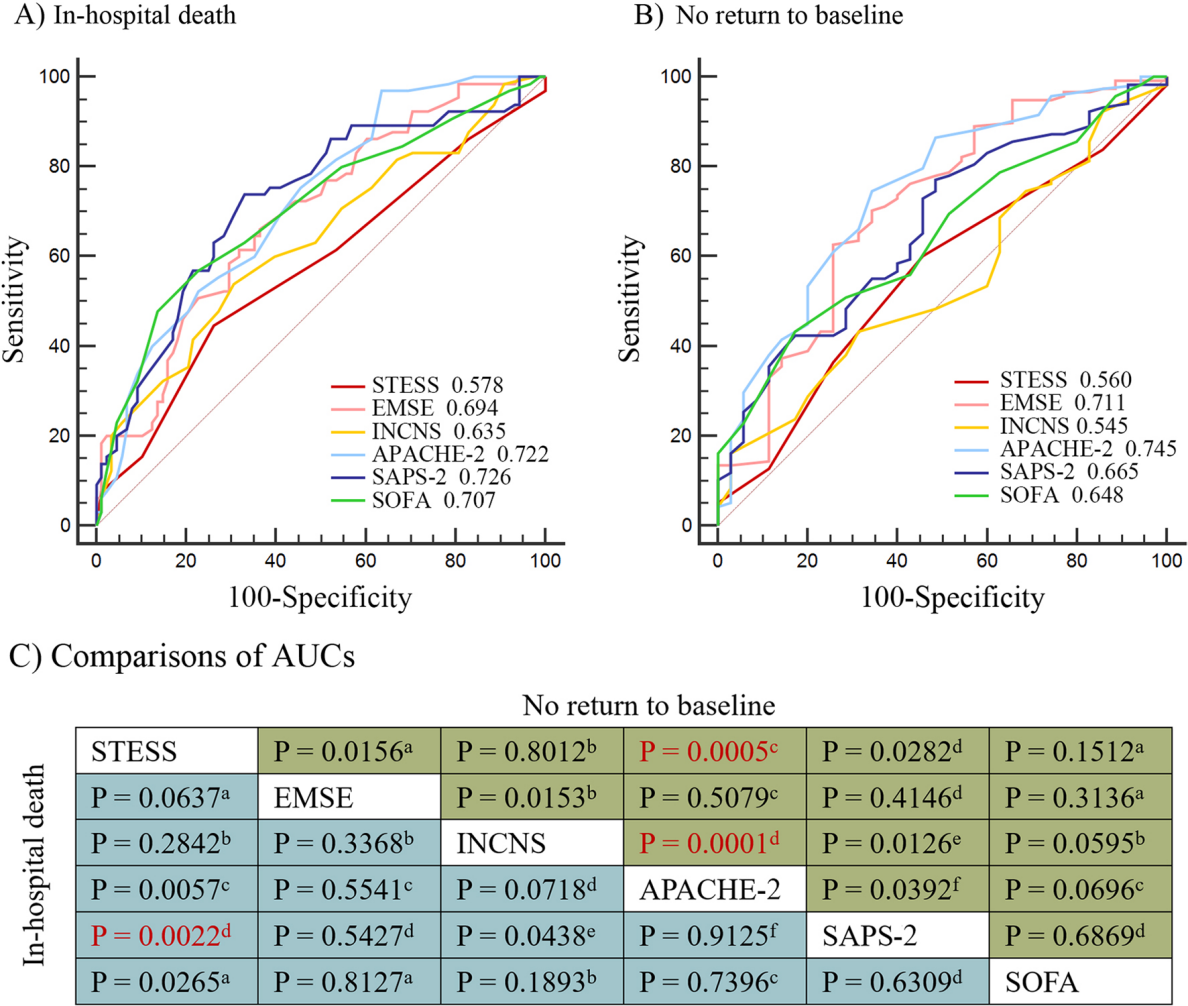
Comparisons of areas under the receiver operating characteristic curves. **A** ROC curves for predicting in-hospital death; **B** ROC curves for predicting no return to baseline status; **C** Pair-wise comparisons of AUCs among six scores. The significance level is 0.0033, and the significant difference is in red. ^a^In 166 patients. ^b^In 162 patients. ^c^In 161 patients. ^d^In 158 patients. ^e^In 155 patients. ^f^In 156 patients

The measurements of the six severity scores are presented in Additional file 1: Table S1. For the prediction of in-hospital death, SAPS-2 had the highest AUC (0.73), while STESS had the lowest AUC (0.58) (Fig. 2A). There was a significant difference between STESS and SAPS-2 (*P* = 0.002) (Fig. 2C). The second highest AUC was for the APACHE-2 (0.72), and the second lowest was for the INCNS (0.64). For the prediction of no return to baseline function, the AUC of APACHE-2 was the highest (0.75) and the AUC of INCNS was the lowest (0.55) (Fig. 2B). The difference between them was significant (*P* < 0.001) (Fig. 2C). The second highest was or the EMSE (0.71), and the second lowest was for the STESS (0.56). The difference of AUC was significant between APACHE-2 and STESS (*P* = 0.0005) (Fig. 2C).

**Fig. 3:**
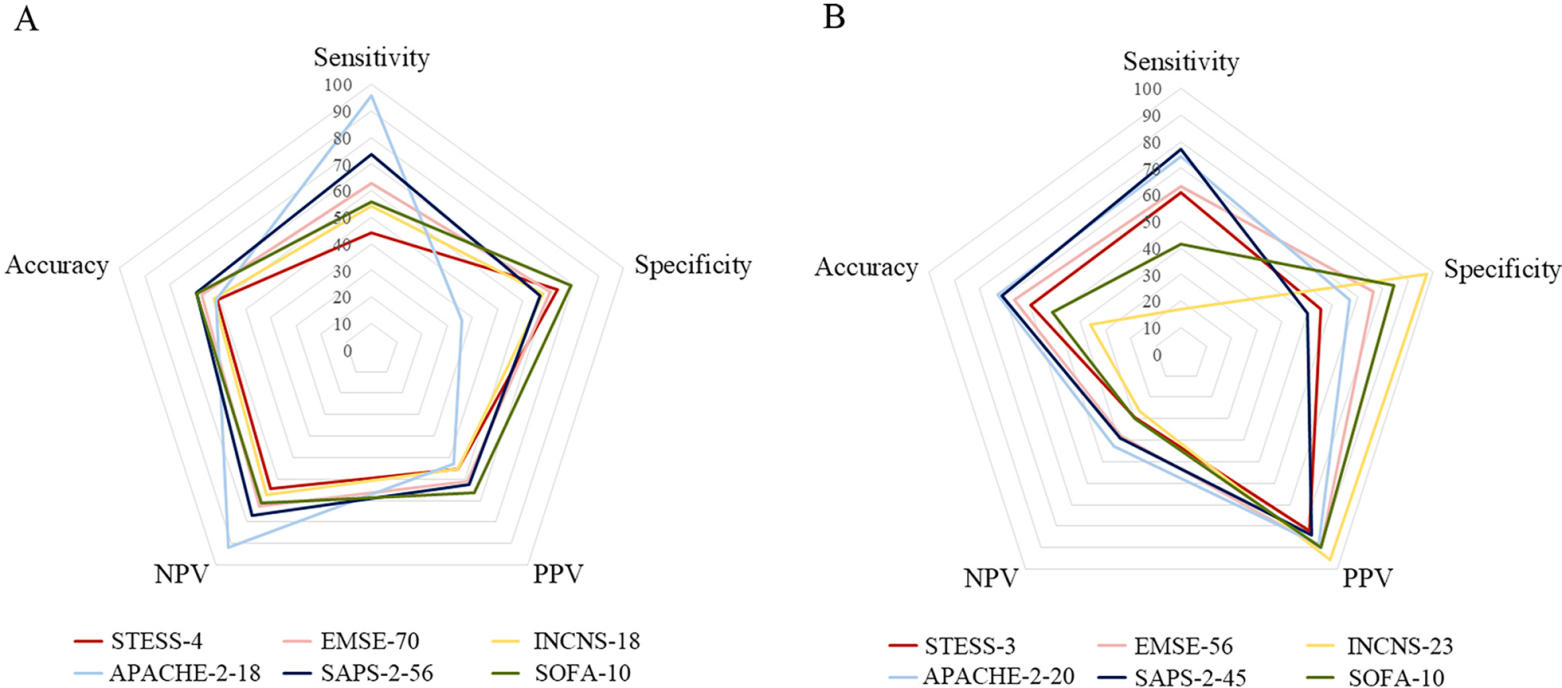
Sensitivity, specificity, PPV, NPV, and accuracy of six scores. **A** Predictive performance for in-hospital death of six scores at their respective best cutoff values. **B** Predictive performance for no return to baseline functional status of six scores at their respective best cutoff values

The sensitivity, specificity, PPV, NPV, accuracy of these six scores with best cutoff values are presented in Fig. 3. For the prediction of in-hospital death, APACHE-2 with the best cutoff value of 18 (APACHE-2-18) had the highest sensitivity (95.7%), and SOFA-10 had the highest specificity (79.2%) (Fig. 3A, Additional file 1: Table S2). SOFA-10 was higher than STESS-4 in all five predictive parameters. For the prediction of no return to baseline, the sensitivity of SAPS-2-45 was the highest (77.1%), and the specificity of INCNS-23 was the highest (97.4%) (Fig. 3B, Additional file 1: Table S3). EMSE-56 and APACHE-2-20 were higher than STESS-3 in all five predictive parameters. The detailed comparisons among the six scores are shown in Additional file 1: Table S4–8. At cutoff values yielding a specificity close to 90%, the sensitivity values of all these scores were all < 40%, both for in-hospital death and no return to baseline (Additional file 1: Table S9–10).

The ROC curves of GCS and systemic severity scores without the GCS (modified APACHE-2, modified SAPS-2, and modified SOFA) are presented in Additional file 1: Figure S1. For the prediction of both in-hospital death and no return to baseline function, all these modified systemic severity scores achieved higher, although mostly non-significantly, AUCs than STESS (Additional file 1: Figure S1). EMSE had a marginally lower AUC (0.69) than modified SAPS-2 (0.71) and modified SOFA (0.70) in predicting in-hospital death, and EMSE had a similar AUC (0.71) to modified APACHE-2 (0.73) in predicting no return to baseline functional status (Additional file 1: Figure S1).

Logistic regression analyses of STESS, EMSE, and systemic severity scores are presented in Table 2. In the univariate analyses, STESS was not significantly associated with in-hospital death or no return to baseline status, and INCNS and GCS scores were not significantly associated with no return to baseline status. EMSE and all the systemic severity scores, with or without the exclusion of GCS, were significantly associated with both in-hospital mortality and no return to baseline status. Multivariate logistic regression analyses showed that the best combinations of scores all included at least one systemic severity scores (with or without the exclusion of GCS).

**Table 2:** Logistic regression analyses for the predictive value of STESS, EMSE, and systemic severity scores

	Univariate		Multivariate^a^	
	OR (95% CI)	*p* value	OR (95% CI)	*p* value
*Predicting in-hospital death*				
Model 1				
STESS	1.241 (0.986–1.561)	0.066		
EMSE	1.025 (1.014–1.037)	< 0.001	1.018 (1.006–1.030)	0.004
INCNS	1.084 (1.024–1.147)	0.006		
APACHE-2	1.142 (1.075–1.213)	< 0.001		
SAPS-2	1.057 (1.032–1.082)	< 0.001	1.048 (1.022–1.075)	< 0.001
SOFA	1.257 (1.137–1.390)	< 0.001		
Model 2				
STESS	1.241 (0.986–1.561)	0.066		
EMSE	1.025 (1.014–1.037)	< 0.001	1.015 (1.003–1.028)	0.013
GCS	0.886 (0.804–0.977)	0.016		
Modified APACHE-2	1.114 (1.047–1.185)	0.001		
Modified SAPS-2	1.079 (1.043–1.117)	< 0.001	1.045 (1.004–1.087)	0.032
Modified SOFA	1.266 (1.138–1.409)	< 0.001	1.137 (1.003–1.289)	0.045
*Predicting no return to baseline*				
Model 1				
STESS	1.185 (0.903–1.556)	0.221		
EMSE	1.030 (1.015–1.045)	< 0.001	1.016 (1.001–1.032)	0.041
INCNS	1.035 (0.972–1.102)	0.284		
APACHE-2	1.192 (1.098–1.295)	< 0.001	1.147 (1.050–1.253)	0.002
SAPS-2	1.037 (1.011–1.064)	0.006		
SOFA	1.179 (1.049–1.324)	0.006		
Model 2				
STESS	1.185 (0.903–1.556)	0.221		
EMSE	1.030 (1.015–1.045)	< 0.001		
GCS	0.935 (0.843–1.037)	0.205	0.877 (0.777–0.991)	0.035
Modified APACHE-2	1.188 (1.090–1.295)	< 0.001	1.210 (1.103–1.327)	< 0.001
Modified SAPS-2	1.066 (1.026–1.108)	0.001		
Modified SOFA	1.197 (1.052–1.362)	0.006		

^a^Stepwise

## Discussion

This study compared the prognostic performance of two SE scores (STESS and EMSE) and four broader critical illness or neurocritical illness scores (INCNS, APACHE-2, SAPS-2, and SOFA) in critically ill patients diagnosed with SE for both mortality and return to baseline functional status. In line with prior evidence [14–16], we found that the STESS lacked accuracy for outcome prediction in critically ill patients with SE and that the EMSE was marginally better than the STESS. In seeming contradiction with prior evidence [21], however, we also found that the APACHE-2 and SAPS-2, as well as their modified versions without the GCS, had better discriminative power for death and functional outcome, respectively, than the STESS. However, none of the investigated scores had sufficient accuracy to be used for clinical decision in isolation at the individual level.

To date, as many as ten prognostic scores have been developed for adult patients with SE: STESS [12], two modified scores based on STESS (mSTESS [32] and nSTESS [33]), EMSE score [13], ENDIT score [34], a new score combining STESS and ENDIT [35], two Thailand scores predicting short- and long-term outcomes based on the national SE database [36, 37], Complication Burden Index (CBI) [38], and Age Consciousness Duration (ACD) score [39]. STESS and EMSE scores are the most widely used scores for status epilepticus and include quite different parameters. Therefore, we chose STESS and EMSE scores in this study and compared these two most representative SE-specific scores with general illness severity scoring systems.

The STESS was the first severity score and the most widely used score for SE [12]. However, a study about SE in North-American ICU reported an AUC of 0.61 for STESS in predicting in-hospital death [15], and a study including patients with refractory SE showed an AUC of 0.57 [14]. STESS made false predictions for death in 51.4% of SE patients, and comorbidity was indicated as a risk factor to be considered [17]. Our study also shows that the prognostic performance of STESS was poor for both mortality and no return to baseline status. Although the STESS was previously shown to be as strongly associated with outcomes as critical illness severity scores in ICU patients with SE [21], our study is the first to assess and compare their respective prognostic accuracy. Compared to this prior study, patients in our study were more severely ill, as attested by higher CCI, APACHE-2, SAPS-2, and SOFA scores and a higher mortality rate, providing a more diverse and possibly more representative cohort of critically ill patients.

There are several versions of EMSE based on different combinations of etiology, comorbidity, level of consciousness, duration, and EEG. EMSE-EACE, including etiology, age, comorbidities, and EEG findings, is the most widely used and most robust version and was investigated in this study. Compared to STESS, the prognostic performance of EMSE was marginally superior. However, it is impossible to obtain an EMSE score on admission as with the STESS score, because some time is needed to determine the SE causes. In the multivariate logistic regression analyses, all the best combinations of scores included at least one or more systemic severity scores. This suggests that while the etiology and preexisting comorbidities assessed by the EMSE-EACE can reflect the vulnerability of SE patients to later multi-organ failures, the assessment of acute systemic complications may provide more accurate information for outcome prediction.

Indeed, without including any parameters specific to SE beyond the GCS, APACHE-2, SAPS-2, and SOFA showed comparable, and in some cases better, prognostic performance than STESS and EMSE. Even the SOFA score, which contains only 6 items for multiple organ (dys)function and is much simpler than other systemic severity scores, achieved a marginally better predictive performance than the STESS for both in-hospital death and no return to baseline condition. After excluding the effect of the GCS, the modified APACHE-2, modified SAPS-2, and modified SOFA still showed a discriminative power greater than the STESS, and comparable to EMSE.

Altogether, these findings indicate that acute systemic comorbidities affect outcome in this population, explaining why STESS and, to a lesser extent, EMSE-EACE are insufficient for outcome prediction for critically ill patients with SE. Systemic severity scores should thus be considered for outcome prediction for critically ill patients with SE, both for clinical and research purposes.

However, none of the scores, be they SE-specific or systemic, had sufficient prognostic power (all AUC < 0.75) when used in isolation. For specificity close to 90%, the sensitivity of all these scores with the respective cutoff values was very low (all < 40%). They are thus not sufficiently reliable to make clinical decisions on limitations of treatment or guide the design of clinical studies. Further studies should thus investigate how best to combine these scores or aim to develop new, more comprehensive, scores for SE patients in ICU.

The study is limited by its retrospective design, although patients were prospectively identified and collected data were readily available from the medical records. The single-center design is also a limitation to the generalizability of our findings, and more prospective multi-centric studies should be conducted to validate these findings. Also, as stated above, we included a cohort of severely ill patients. In addition, lack of return to baseline functional status may not be a precise indicator of the impact of SE and we analyzed it only for the sake of comparison with prior studies. A patient whose mRS score remains stable at 3 is still more severely impaired than a patient whose mRS score rises from 0 to 1, and a patient with a stable mRS score of 3 may also have a significant decline in functional status.

## Conclusions

Both STESS and EMSE, two severity scores specifically designed for SE, were insufficient in outcome prediction for critically ill patients with SE, and EMSE was marginally better than STESS. INCNS score, a severity score for neurocritical illnesses, also had inadequate predictive power, whereas APACHE-2, SAPS-2, and SOFA score achieved better performance. None of these scores have the desirable prognostic power to be used in clinical practice in isolation. However, the combinations of both EMSE and systemic severity scores offered the best prognostic models. Systemic illness thus matters in ICU patients with SE, and SE scores should be modified to achieve better accuracy in this severely ill population. This study mostly refers to severely ill patients in the ICU.

## Supplementary Information

**Additional file 1:** Table S1–S10 and Figure S1.

## Data Availability

The datasets used and analyzed during the current study are available from the corresponding author on reasonable request.
