# Supplementary material for: Severity scores for status epilepticus in the ICU: systemic illness also matters
Source: Crit Care. 2023 Jan 16;27:19. doi: 10.1186/s13054-022-04276-7 (PMC9841666; doi:10.1186/s13054-022-04276-7)
Supplement: Supplementary file 1 — Additional file 1: Table S1–S10 and Figure S1. [file 13054_2022_4276_MOESM1_ESM.docx]

Supplementary materials

1. Table S1. Parameters of six severity scores.
2. Table S2. Sensitivity, specificity, PPV, NPV, and accuracy for the prediction of in-hospital death.
3. Table S3. Sensitivity, specificity, PPV, NPV, and accuracy for the prediction of no return to baseline.
4. Table S4. Comparisons of sensitivity.
5. Table S5. Comparisons of specificity.
6. Table S6. Comparisons of positive predive value.
7. Table S7. Comparisons of negative predictive value.
8. Table S8. Comparisons of accuracy.
9. Table S9. Sensitivity for the prediction of in-hospital death at cutoff values yielding a specificity close to 90%.
10. Table S10. Sensitivity for the prediction of no return to baseline at cutoff values yielding a specificity close to 90%.
11. Figure S1. ROC curves of STESS, EMSE, GCS, modified APACHE 2, modified SAPS 2, modified SOFA.

**Table S1. Measurements of six severity scores.**

| Parameters | STESS | EMSE-EACE | INCNS | APACHE-2 | SAPS-2 | SOFA |
| --- | --- | --- | --- | --- | --- | --- |
| Consciousness | Alert or somnolent/ confused; stuporous or comatose | - | Arousal and awareness | GCS | GCS | GCS |
| Worst seizure type | SP or CP; GC; NCSEC | - | - | - | - | - |
| Age | <65 or ≥ 65 years | 7 categories | 4 categories | 5 categories | 6 categories | - |
| History of seizures | Yes | - | - | - | - | - |
| Etiology | - | 15 categories | - | Elective / emergency surgery or not | Elective / emergency surgery or not | - |
| Comorbidity | - | Yes | - | - | Yes | - |
| Electroencephalography | - | Yes | - | - | - | - |
| Brainstem reflexes | - | - | Pupillary and corneal | - | - | - |
| Verbal and motor responses | - | - | Yes | GCS | GCS | GCS |
| Swallowing function | - | - | Yes | - | - | - |
| Respiration rate | - | - | Yes | Yes | - | - |
| Heart rate | - | - | Yes | Yes | Yes | - |
| Blood pressure | - | - | SBP | MAP | SBP | MAP |
| Temperature | - | - | Yes | Yes | Yes | - |
| White blood cells | - | - | Yes | Yes | Yes | - |
| Blood glucose | - | - | Yes | - | - | - |
| Serum albumin | - | - | Yes | - | - | - |
| Serum sodium | - | - | Yes | Yes | Yes | - |
| Serum creatinine | - | - | Yes | Yes | - | Yes |
| Serum bilirubin | - | - | Yes | - | Yes | Yes |
| Oxygenation | - | - | - | Yes | Yes | Yes |
| Arterial PH | - | - | - | Yes | - | - |
| Bicarbonate | - | - | - | Yes | Yes | - |
| Serum potassium | - | - | - | Yes | Yes | - |
| Hematocrit | - | - | - | Yes | - | - |
| Urinary output | - | - | - | - | Yes | Yes |
| Blood urea nitrogen | - | - | - | - | Yes | - |
| Platelets | - | - | - | - | - | Yes |

CP, complex partial; GC, generalized convulsive; GCS, Glasgow coma scale; MAP, mean arterial pressure; NCSEC, nonconvulsive status epilepticus with coma; SP, simple partial; SBP, systolic blood pressure.

**Table S2. Sensitivity, specificity, PPV, NPV, and accuracy for the prediction of in-hospital death.**

| Scores | Sensitivity | Specificity | PPV | NPV | Accuracy |
| --- | --- | --- | --- | --- | --- |
| STESS ≥ 4 | 44.3% | 74.0% | 55.4% | 64.6% | 61.5% |
| EMSE ≥ 70 | 62.9% | 70.8% | 61.1% | 72.3% | 67.5% |
| INCNS ≥ 18 | 54.4% | 68.1% | 55.2% | 67.4% | 62.4% |
| APACHE Ⅱ ≥ 18 | 95.7% | 35.9% | 52.8% | 91.7% | 61.5% |
| SAPS Ⅱ ≥ 56 | 73.5% | 66.7% | 62.5% | 76.9% | 69.6% |
| SOFA ≥ 10 | 55.7% | 79.2% | 66.1% | 71.0% | 69.3% |

PPV, positive predictive value; NPV, negative predictive value.

**Table S3. Sensitivity, specificity, PPV, NPV, and accuracy for the prediction of no return to baseline.**

| Scores | Sensitivity | Specificity | PPV | NPV | Accuracy |
| --- | --- | --- | --- | --- | --- |
| STESS ≥ 3 | 60.9% | 55.3% | 82.1% | 29.6% | 59.6% |
| EMSE ≥ 56 | 63.3% | 76.3% | 90.0% | 38.2% | 66.3% |
| INCNS ≥ 23 | 17.1% | 97.4% | 95.5% | 26.6% | 36.0% |
| APACHE Ⅱ ≥ 20 | 74.4% | 66.7% | 88.6% | 42.9% | 72.7% |
| SAPS Ⅱ ≥ 45 | 77.1% | 50.0% | 83.9% | 39.1% | 70.9% |
| SOFA ≥ 10 | 41.4% | 84.2% | 89.8% | 29.9% | 51.2% |

PPV, positive predictive value; NPV, negative predictive value.

**Table S4. Comparisons of Sensitivity*.**

|  | No return to baseline | | | | | |
| --- | --- | --- | --- | --- | --- | --- |
| In-hospital death | STESS | P = 0.7914^a^ | P < 0.0001^b^ | P = 0.0175^c^ | P = 0.0019^d^ | P = 0.0026^a^ |
|  | P = 0.0410^a^ | EMSE | P < 0.0001^b^ | P = 0.0444^c^ | P = 0.0114^d^ | P = 0.0003^a^ |
|  | P = 0.2863^b^ | P = 0.3915^b^ | INCNS | P < 0.0001^d^ | P < 0.0001^e^ | P = 0.0001^b^ |
|  | P < 0.0001^c^ | P < 0.0001^c^ | P < 0.0001^d^ | APACHE-2 | P = 0.8555^f^ | P < 0.0001^c^ |
|  | P = 0.0002^d^ | P = 0.1849^d^ | P = 0.0106^e^ | P < 0.0001^f^ | SAPS-2 | P < 0.0001^d^ |
|  | P = 0.2153^a^ | P = 0.4731^a^ | P = 1.0000^b^ | P < 0.0001^c^ | P = 0.0192^d^ | SOFA |

*The significance level is 0.0033. ^a^In 166 patients. ^b^In 162 patients. ^c^In 161 patients. ^d^In 158 patients. ^e^In 155 patients. ^f^In 156 patients.

**Table S5. Comparisons of Specificity*.**

|  | No return to baseline | | | | | |
| --- | --- | --- | --- | --- | --- | --- |
| In-hospital death | STESS | P = 0.0574^a^ | P = 0.0001^b^ | P = 0.3877^c^ | P = 0.7539^d^ | P = 0.0074^a^ |
|  | P = 0.7283^a^ | EMSE | P = 0.0215^b^ | P = 0.5488^c^ | P = 0.0225^d^ | P = 0.5488^a^ |
|  | P = 0.4583^b^ | P = 0.8714^b^ | INCNS | P = 0.0010^d^ | P < 0.0001^e^ | P = 0.0625^b^ |
|  | P < 0.0001^c^ | P < 0.0001^c^ | P < 0.0001^d^ | APACHE-2 | P = 0.1797^f^ | P = 0.1094^c^ |
|  | P = 0.1892^d^ | P = 0.8642^d^ | P = 0.6900^e^ | P < 0.0001^f^ | SAPS-2 | P = 0.0005^d^ |
|  | P = 0.4583^a^ | P = 0.2295^a^ | P = 0.1102^b^ | P < 0.0001^c^ | P = 0.0639^d^ | SOFA |

*The significance level is 0.0033. ^a^In 166 patients. ^b^In 162 patients. ^c^In 161 patients. ^d^In 158 patients. ^e^In 155 patients. ^f^In 156 patients.

**Table S6. Comparisons of Positive predive value*.**

|  | No return to baseline | | | | | |
| --- | --- | --- | --- | --- | --- | --- |
| In-hospital death | STESS | P = 0.0290^a^ | P = 0.0199^b^ | P = 0.0633^c^ | P = 0.5120^d^ | P = 0.0723^a^ |
|  | P = 0.3823^a^ | EMSE | P = 0.2695^b^ | P = 0.7321^c^ | P = 0.0821^d^ | P = 0.9681^a^ |
|  | P = 0.9821^b^ | P = 0.3958^b^ | INCNS | P = 0.1265^d^ | P = 0.0397^e^ | P = 0.1937^b^ |
|  | P = 0.6058^c^ | P = 0.0894^c^ | P = 0.4418^d^ | APACHE-2 | P = 0.1280^f^ | P = 0.7679^c^ |
|  | P = 0.2750^d^ | P = 0.6503^d^ | P = 0.3360^e^ | P = 0.0092^f^ | SAPS-2 | P = 0.0807^d^ |
|  | P = 0.1192^a^ | P = 0.4386^a^ | P = 0.1049^b^ | P = 0.0145^c^ | P = 0.4766^d^ | SOFA |

*The significance level is 0.0033. ^a^In 166 patients. ^b^In 162 patients. ^c^In 161 patients. ^d^In 158 patients. ^e^In 155 patients. ^f^In 156 patients.

**Table S7. Comparisons of Negative predictive value*.**

|  | No return to baseline | | | | | |
| --- | --- | --- | --- | --- | --- | --- |
| In-hospital death | STESS | P = 0.0752^a^ | P = 0.3428^b^ | P = 0.0099^c^ | P = 0.0703^d^ | P = 0.9369^a^ |
|  | P = 0.0701^a^ | EMSE | P = 0.0009^b^ | P = 0.2335^c^ | P = 0.7170^d^ | P = 0.0330^a^ |
|  | P = 0.5247^b^ | P = 0.2831^b^ | INCNS | P = 0.0003^d^ | P = 0.0142^e^ | P = 0.0693^b^ |
|  | P < 0.0001^c^ | P = 0.0014^c^ | P < 0.0001^d^ | APACHE-2 | P = 0.4615^f^ | P = 0.0038^c^ |
|  | P = 0.0016^d^ | P = 0.1964^d^ | P = 0.0183^e^ | P = 0.0008^f^ | SAPS-2 | P = 0.0868^d^ |
|  | P = 0.0940^a^ | P = 0.7600^a^ | P = 0.3487^b^ | P = 0.0002^c^ | P = 0.1046^d^ | SOFA |

*The significance level is 0.0033. ^a^In 166 patients. ^b^In 162 patients. ^c^In 161 patients. ^d^In 158 patients. ^e^In 155 patients. ^f^In 156 patients.

**Table S8. Comparisons of Accuracy*.**

|  | No return to baseline | | | | | |
| --- | --- | --- | --- | --- | --- | --- |
| In-hospital death | STESS | P = 0.6353a | P < 0.0001^b^ | P = 0.1034^c^ | P = 0.0022^d^ | P = 0.0001^a^ |
|  | P = 0.0681^a^ | EMSE | P < 0.0001^b^ | P = 0.0273^c^ | P = 0.0006^d^ | P = 0.0002^a^ |
|  | P = 0.1608^b^ | P = 0.7239^b^ | INCNS | P < 0.0001^d^ | P < 0.0001^e^ | P < 0.0001^b^ |
|  | P < 0.0001^c^ | P < 0.0001^c^ | P < 0.0001^d^ | APACHE-2 | P = 0.3368^f^ | P < 0.0001^c^ |
|  | P = 0.0001^d^ | P = 0.2529^d^ | P = 0.0293^e^ | P < 0.0001^f^ | SAPS-2 | P < 0.0001^d^ |
|  | P = 0.7982^a^ | P = 0.1360^a^ | P = 0.2976^b^ | P < 0.0001^c^ | P = 0.0019^d^ | SOFA |

*The significance level is 0.0033. ^a^In 166 patients. ^b^In 162 patients. ^c^In 161 patients. ^d^In 158 patients. ^e^In 155 patients. ^f^In 156 patients.

**Table S9. Sensitivity for the prediction of in-hospital death at cutoff values yielding a specificity close to 90%.**

| Scores | Specificity (95% CI) | Sensitivity (95% CI) |
| --- | --- | --- |
| STESS ≥ 5 | 88.5% (80.4% - 94.1%) | 14.3% (7.1% - 24.7%) |
| EMSE ≥ 98 | 90.6% (82.9% - 95.6%) | 20.0% (11.4% - 31.3%) |
| INCNS ≥ 23 | 92.5% (85.3% - 97.0%) | 23.5% (14.1% - 35.4%) |
| APACHE Ⅱ ≥ 28 | 90.2% (82.2% - 95.4%) | 33.3% (22.4% - 45.7%) |
| SAPS Ⅱ ≥ 69 | 91.1% (83.2% - 96.1%) | 30.9% (20.2% - 43.3%) |
| SOFA ≥ 12 | 91.7% (84.2% - 96.3%) | 32.9% (22.2% - 45.1%) |

**Table S10. Sensitivity for the prediction of no return to baseline at cutoff values yielding a specificity close to 90%.**

| Scores | Specificity (95% CI) | Sensitivity (95% CI) |
| --- | --- | --- |
| STESS ≥ 5 | 89.5% (75.2% - 97.1%) | 13.3% (7.9% - 20.4%) |
| EMSE ≥ 82 | 89.5% (75.2% - 97.1%) | 33.6% (25.5% - 42.5%) |
| INCNS ≥ 22 | 84.2% (68.7% - 94.0%) | 25.0% (17.7% - 33.6%) |
| APACHE Ⅱ ≥ 26 | 88.9% (73.9% - 96.9%) | 37.6% (29.1% - 46.7%) |
| SAPS Ⅱ ≥ 67 | 88.9% (73.9% - 96.9%) | 27.9% (20.1% - 36.7%) |
| SOFA ≥ 11 | 89.5% (75.2% - 97.1%) | 32.0% (24.1% - 40.9%) |

Figure S1. ROC curves of STESS, EMSE, GCS, modified APACHE 2, modified SAPS 2, modified SOFA.*


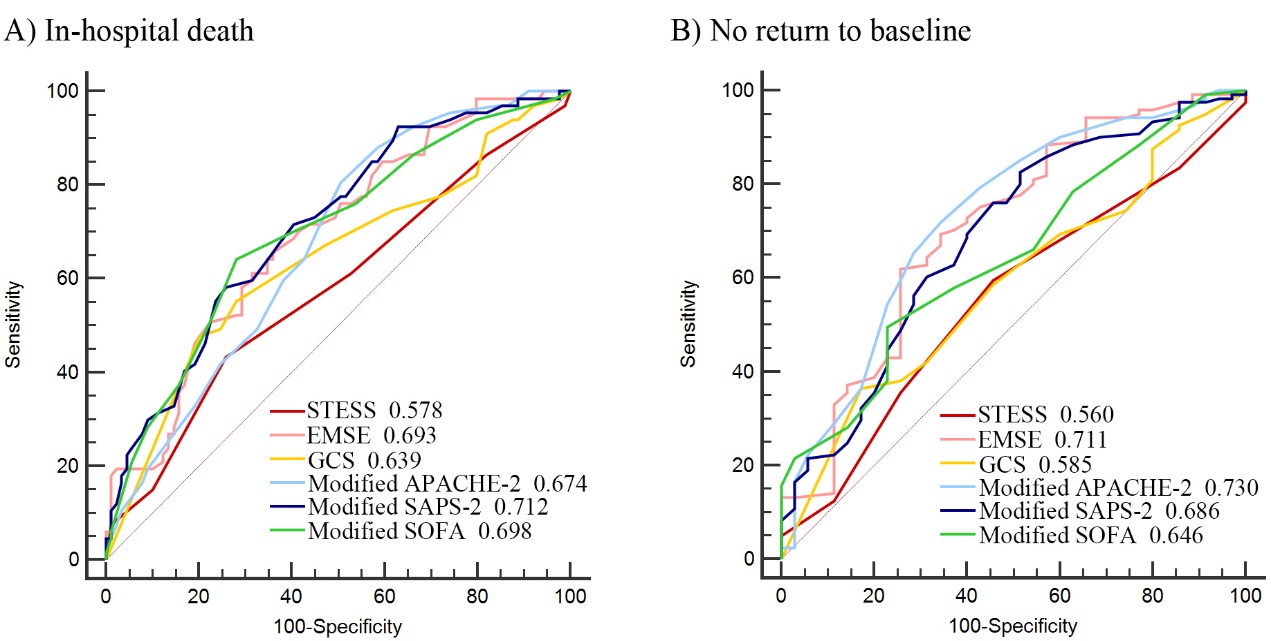


*Modified APACHE-2: APACHE-2 without GCS. Modified SAPS-2: SAPS-2 without GCS. Modified SOFA: SOFA without GCS. For the prediction of in-hospital death, GCS ≤ 3, modified APACHE-2 ≥ 12, modified SAPS-2 ≥ 43, SOFA ≥ 6 are best cut-off values determined by the Youden index. For the prediction of no return to baseline status, GCS ≤ 3, modified APACHE-2 ≥ 12, modified SAPS-2 ≥ 31, SOFA ≥ 6 are best cut-off values determined by the Youden index.
